# EpuDB: an integrated functional genomics database and REST API for *Epimedium pubescens*

**DOI:** 10.3389/fpls.2026.1967816

**Published:** 2026-10-27

**Authors:** Jiaotong Yang, Siran Wang, Mingxian Zhou, Yaxin Shuai, Qingwen Sun

**Affiliations:** Resource Institute for Chinese and Ethnic Materia Medica, Guizhou University of Traditional Chinese Medicine, Guiyang, Guizhou, China

**Keywords:** co-expression network, differential expression, *Epimedium pubescens*, functional genomics database, icariin, prenylated flavonoids, REST API, transcriptome

## Abstract

**Introduction:**

*Epimedium pubescens Maxim.* (Berberidaceae) is a botanical source of Herba Epimedii and is rich in prenylated flavonoids such as icariin. Although a chromosome-level genome is available for *E. pubescens*, its genomic and transcriptomic resources are dispersed across public resource platforms. We developed EpuDB (https://www.gzybioinformatics.cn/EpuDB/) to bring these resources together in a searchable and programmatically accessible database.

**Methods:**

EpuDB links 42,827 official gene loci to functional annotations, gene-family classifications, transcriptomic profiles, differential-expression results, co-expression networks and orthology-inferred candidate protein–protein interactions.

**Results:**

The database contains 1,991 transcription factors, 521 transcriptional regulators, 1,139 protein kinases, 505 cytochrome P450s and 1,106 ubiquitination-system components, together with 2,399 candidate protein–protein interaction pairs. Its transcriptome compendium includes 70 RNA-seq samples from six BioProjects and 58 within-project differential-expression contrasts, yielding 59,713 significant differentially expressed gene records. The historical and BioProject-adjusted co-expression networks contain 358,077 and 134,840 positive edges, respectively, and 73,411 and 171,368 negative edges, respectively. In an icariin-biosynthesis case study, three representative loci were prioritized: *Ebr01G061770* and *Ebr01G061810* as candidate O-methyltransferases, and *Ebr05G059870* as a candidate R2R3-MYB transcriptional regulator associated with prenyltransferase genes.

**Discussion:**

Collectively, the domain, expression and co-expression evidence for these loci provides a testable hypothesis for 4′-O-methylation and for transcriptional regulation, respectively. EpuDB provides a traceable starting point for gene discovery and hypothesis generation in *E. pubescens*.

## 1 Introduction

*Epimedium pubescens Maxim.* is a perennial herb in the family Berberidaceae and a botanical source of Herba Epimedii (Shen et al., 2022). Herba Epimedii has long been used in traditional Chinese medicine. Its major constituents include prenylated flavonol glycosides and related aglycones, such as icariin, epimedin A, epimedin B, epimedin C and icaritin (Wang et al., 2016; Li et al., 2021). These compounds have been studied in relation to bone metabolism, inflammation, neuroprotection and vascular biology (Sze et al., 2010; Li et al., 2021).

The biosynthetic basis of prenylflavonoid production in *Epimedium* was still incomplete when Shen et al. reported a chromosome-level genome assembly for *E. pubescens* (Shen et al., 2022). The assembly combined Oxford Nanopore long reads, Illumina polishing and Hi-C scaffolding to produce six pseudochromosomes, consistent with the diploid karyotype 2n = 2x = 12. The study also identified candidate flavonoid prenyltransferases and functionally characterized EpPT8 as an enzyme acting on icaritin-related substrates (Shen et al., 2022).

The genome provided a structural foundation for *E. pubescens*, but not a continuously queryable resource that linked annotation, gene families, expression, networks and analysis tools. Resources such as MaGenDB and PLAZA illustrate the value of combining genome annotation with multi-omics data and common query interfaces (Wang et al., 2020; Van Bel et al., 2018). A comparable resource would help organize *E. pubescens* candidates distributed among prenyltransferases, cytochrome P450s, glycosyltransferases, transcription factors and transporters.

Here we present EpuDB, an integrated functional genomics database and REST API for E. pubescens. EpuDB reorganizes the official gene catalogue from assembly GWHBECS00000000 and links it to functional annotations, gene-family catalogues, interaction and co-expression networks, transcripts per million (TPM) profiles and differential-expression results. The web portal and JSON API expose the same evidence layers for interactive browsing and programmatic retrieval.

## 2 Materials and methods

### 2.1 Genome data source

We built EpuDB from the chromosome-level *E. pubescens* assembly reported by Shen et al. (Shen et al., 2022) and deposited in the NGDC Genome Warehouse (Chen et al., 2021) under accession GWHBECS00000000 (BioProject PRJCA006303). We imported the accompanying official gene models without reannotation. Cross-reference tables linked gene, transcript and protein identifiers across genomic, transcriptomic and functional-annotation datasets. We parsed coordinates, strand information and feature relationships from the accompanying GFF3 file, then sorted and indexed the file for JBrowse2 visualization (Diesh et al., 2023).

### 2.2 Functional annotation

We used DIAMOND blastp (Buchfink et al., 2021) in best-hit mode (--top 1) against NCBI NR, Swiss-Prot, the Arabidopsis thaliana TAIR10 proteome and TrEMBL. The E-value cut-off was 1 × 10^-5^. We generated Gene Ontology and Pfam annotations with InterProScan (Jones et al., 2014). We assigned KEGG Orthology and pathway annotations with KofamScan (Aramaki et al., 2020), using profile HMM searches and linking KO assignments to enzyme identifiers and KEGG pathway map IDs.

### 2.3 Gene-family catalogues

#### 2.3.1 Regulatory and signaling gene families

We generated seven gene-family catalogues. iTAK v2.0.7 classified transcription factors, transcriptional regulators and protein kinases (Zheng et al., 2016). We identified cytochrome P450 monooxygenases with HMMER 3.4 against Pfam PF00067.28 using the curated gathering threshold (--cut_ga). We identified ubiquitination-system components with HMMER 3.4 and the iUUCD2 HMM collection, applying full-sequence and domain E-value cut-offs of 1 × 10^-5^ (Zhou et al., 2018).

#### 2.3.2 CAZymes and CAZyme-associated proteins

We analyzed 42,813 translated proteins from the 42,827 official gene models. dbCAN 5.2.9 (Zheng et al., 2023) supplied three evidence streams: DIAMOND against CAZy, pyHMMER against dbCAN family HMMs and pyHMMER against dbCAN-sub. The DIAMOND search used an E-value cut-off of 1 × 10^-102^ and one target. The HMM searches used E-value ≤ 1 × 10^-15^ and HMM coverage ≥ 0.35. We called a family high confidence only when the same CAZy family was supported by at least two methods. Single-method calls and discordant multi-method calls were retained as candidates. GH, GT, PL, CE and AA assignments were counted as catalytic CAZymes; CBM-only proteins were classified as CAZyme-associated proteins.

#### 2.3.3 Transporter-system proteins

We searched the same proteome against a versioned TCDB sequence snapshot (Saier et al., 2021) containing 24,281 sequences with DIAMOND 2.1.13. Discovery hits required E-value ≤ 1 × 10^-4^ and query coverage ≥ 35%. The high-confidence sequence tier required E-value ≤ 1 × 10^-10^, identity ≥ 30%, query and reference coverage ≥ 50%, TCDB class 1–5 and family agreement among near-best hits with bit score ≥ 95% of the best hit. TMbed 1.0.2 (Bernhofer and Rost, 2022) provided independent topology support; signal peptides were not counted as transmembrane segments. Concordant query and reference topology defined core membrane transporters. Concordant hits without a predicted transmembrane segment were labelled soluble or peripheral transporter-system components.

### 2.4 Orthology-inferred candidate protein–protein interaction network

To build an orthology-inferred candidate protein–protein interaction (PPI) network, we filtered *Arabidopsis thaliana* BioGRID (Stark et al., 2006) and IntAct/IMEx records (Kerrien et al., 2012) to unique TAIR gene pairs supported by at least two publications or experimental methods, excluding genetic, negative, self, non-protein, colocalization and proximity-only records. We then mapped Arabidopsis proteins to *E. pubescens* proteins by bidirectional BLASTP and retained strict one-to-one reciprocal best hits with E-value ≤ 1 × 10^-10^, amino-acid identity ≥ 30% and ≥ 50% coverage in both directions; tied hits were excluded. Interactions were transferred only when both partners passed the mapping, and duplicate undirected edges and self-interactions were removed. Co-expression data were not used to construct or filter this network.

### 2.5 Transcriptome compendium, expression matrix and differentially expressed gene analysis

The transcriptome compendium comprised 70 RNA-seq samples from six public BioProjects spanning developmental, tissue and treatment contexts relevant to *Epimedium* biology (**Table 1**). Ten contrasts contained fewer than three libraries in one or both groups and were retained as power-limited analyses. We calculated annotation coverage against 42,827 official *E. pubescens* loci. A locus counted once for a source when at least one linked record was present, regardless of the number of records. We reported RNA-seq mapping and gene-detection rates for every sample and summarized them within each BioProject using medians and full ranges.

**Table 1:** RNA-seq BioProjects integrated in EpuDB.

BioProject	Samples	Description
DRP015081	9	Transcriptome of pod/fruit abscission in *E. pubescens*
SRP329948	11	*E. pubescens* cultivar “Guirou 1” multi-tissue transcriptome associated with the genome project
SRP492263	12	Dynamic adaptive transcriptional responses of *E. pubescens* to phosphorus deficiency
SRP498591	9	*E. pubescens* samples retained from a mixed *Epimedium* RNA-seq BioProject
SRP522167	2	*E. pubescens* samples retained from a multi-species *Epimedium* transcriptome BioProject
SRP523253	27	Transcriptional responses to different nitrogen fertilization levels in *E. pubescens*

We quality-filtered raw RNA-seq reads with fastp (Chen et al., 2018), aligned clean reads to the *E. pubescens* reference genome with HISAT2 (Kim et al., 2019) and estimated gene-level abundance with StringTie (Pertea et al., 2015). For each contrast, a gene was eligible when TPM ≥ 1 in at least min(n_control, n_treatment) libraries. Eligible values were transformed as log_2_(TPM + 1), whereas all 42,827 loci remained in the exported table. Differential expression was analyzed with limma (Ritchie et al., 2015). Limma-trend was applied to log_2_-transformed TPM values with a group-specific design matrix and prespecified contrasts. Models were fitted with lmFit, followed by contrast fitting and empirical Bayes moderation with eBayes(trend = TRUE). P values were adjusted with the Benjamini–Hochberg method. Genes with adjusted P < 0.05 and absolute log_2_ fold change ≥ 1 were classified as differentially expressed. All 58 prespecified contrasts compared groups within the same BioProject-derived experimental series; SRP498591 contrasts used only retained *E. pubescens* libraries. Contrasts with fewer than three libraries in either group were retained and labelled power-limited. For quality control, we parsed the original fastp JSON reports and HISAT2 summaries for every run. We reconciled after-filter read totals with HISAT2 inputs and retained post-filter Q30, read-retention, duplication and overall-alignment rates at sample level. Gene detection was defined as TPM ≥ 1, with TPM > 0 reported as a sensitivity definition. PCA used log_2_(TPM + 1) values for the 5,000 genes with highest median absolute deviation after retaining genes with TPM ≥ 1 in at least three samples. We calculated Spearman sample correlations and average-linkage clusters for all samples. BioProject-associated multivariate structure and dispersion were evaluated by PERMANOVA and PERMDISP with 9,999 permutations. The corresponding RNA-seq quality-control and BioProject-adjusted network audit is provided in **Supplementary Figure S1**.

### 2.6 Co-expression network

A genome-wide co-expression network was computed from the 70-sample gene-level TPM matrix. Pearson correlation coefficients (PCCs) were calculated with the adjacency() function in the WGCNA R package (Langfelder and Horvath, 2008). PCC values were converted to mutual-rank (MR) scores (Obayashi and Kinoshita, 2009). Gene pairs with PCC > 0.7 and MR < 30 were retained as positive edges, whereas pairs with PCC < -0.5 and MR < 30 were retained as negative edges. The historical network combined six BioProjects without cross-project adjustment. Because BioProject was confounded with tissue, stage, treatment, time point and platform, global ComBat correction was not applied. As a sensitivity analysis, log_2_(TPM + 1) values for the same 24,116 eligible genes were centered within each BioProject, and residuals were used to build a separate BioProject-adjusted network with the same PCC and MR thresholds. A matched log-unadjusted network used the identical genes and samples. Adjusted edges were annotated with within-project direction, threshold support, six leave-one-BioProject-out PCCs, zero crossing and the project producing the largest change. Parametric P values and an approximate global BH-FDR were annotations, not edge filters. Threshold sensitivity was evaluated without tuning on a fixed 18-configuration grid: positive PCC cutoffs of 0.60, 0.70 and 0.80 and negative PCC cutoffs of -0.40, -0.50 and -0.60, each crossed with MR cutoffs of 10, 30 and 50. Edge counts, network Jaccard overlap, direction support, leave-one-project-out stability and case-study degree were reported. Project-specific Pearson correlation coefficients were calculated separately from raw TPM and log_2_(TPM + 1) values for DRP015081 (n = 9), SRP329948 (n = 11), SRP492263 (n = 12), SRP498591 (n = 9) and SRP523253 (n = 27); SRP522167 (n = 2) is marked not evaluable. These values are project-specific support and sensitivity annotations, not assignments of biological edge origin.

As a threshold network benchmark, the *E. pubescens* homolog of *EsMYBF1* (Ebr01G065030) was compared with homologs of three published flavonol-pathway targets: F3H (Ebr04G062950), FLS3 (Ebr04G051790) and CHS2 (Ebr05G049130). We reported adjusted PCCs and ranks among 24,116 eligible positive correlations as continuous measures, together with recovery at the prespecified PCC > 0.70 threshold as a binary measure. Project-specific support was summarized across the five evaluable BioProjects, and the threshold was not tuned to the controls.

### 2.7 Web analysis tools

Web-based tools included BLAST searches with ViroBLAST and NCBI BLAST+ (Deng et al., 2007; Camacho et al., 2009), Primer3-based primer design (Untergasser et al., 2012), MUSCLE-based multiple sequence alignment (Edgar, 2004), sequence extraction and GO/KEGG enrichment analysis. Enrichment used a hypergeometric over-representation test with Benjamini–Hochberg FDR correction.

### 2.8 Icariin-biosynthesis candidate prioritization

To demonstrate database use, we defined the icariin pathway from reviewed pathway evidence (Huang et al., 2026). Candidate upstream enzymes were retrieved from KEGG/KO and homology annotations. Five *E. pubescens* O-methyltransferase candidates were audited using PF00891 (Methyltransf_2 O-methyltransferase) and PF08100 (OMT dimerization) architecture, homology, KO annotation, protein length and expression breadth. The three-gene case study retained Ebr01G061770 and Ebr01G061810 as joint leading O-methyltransferase candidates and Ebr05G059870 as a leading R2R3-MYB candidate associated with prenyltransferase genes. The pathway-locus inventory is supplied as **Supplementary Table S1**, and the gene-centered workflow is summarized in **Supplementary Figure S2**.

We selected literature-derived controls spanning same-species retrieval, unresolved locus mapping and cross-species homology. The Results section focuses on EpPT8, Ep7GT, EpPF3RT and EsMYBF1; retrieval status and evidence class were recorded separately in the control table (Shen et al., 2022; Feng et al., 2018, 2019; Huang et al., 2016). The literature-derived controls were analyzed separately from the three-locus icariin case study.

We used pathway candidates as baits in the historical positive co-expression network and intersected their neighbors with the curated transcription-factor catalogue. For each candidate, we reviewed edge-level PCC and MR values, expression coverage, orthology-inferred candidate PPI records and gene-detail annotations in an evidence matrix. Candidates detected in only one sample were excluded from regulatory prioritization. We then evaluated the same case-study pairs in the matched BioProject-adjusted network and recorded project-specific PCC support, threshold recovery and sensitivity annotations.

## 3 Results

### 3.1 Database architecture and REST API

EpuDB separated data storage and service from presentation (**Figure 1**). MySQL stored gene annotations, GO, KEGG, Pfam, gene-family records, strict-RBH candidate PPI edges with source evidence and mapping tables, co-expression edges, the separate BioProject-adjusted sensitivity layer, locus and ID-conversion tables, sequences, sample metadata, contrasts, gene-level TPM values and DEG results. DEG records exposed tested, filter_status, adj_pvalue and neglog10_adj_pvalue fields, while low-expression rows remained available with explicit status fields. The portal used JBrowse2 to display the indexed GFF3 track and linked database records to locus coordinates.

**Figure 1:**
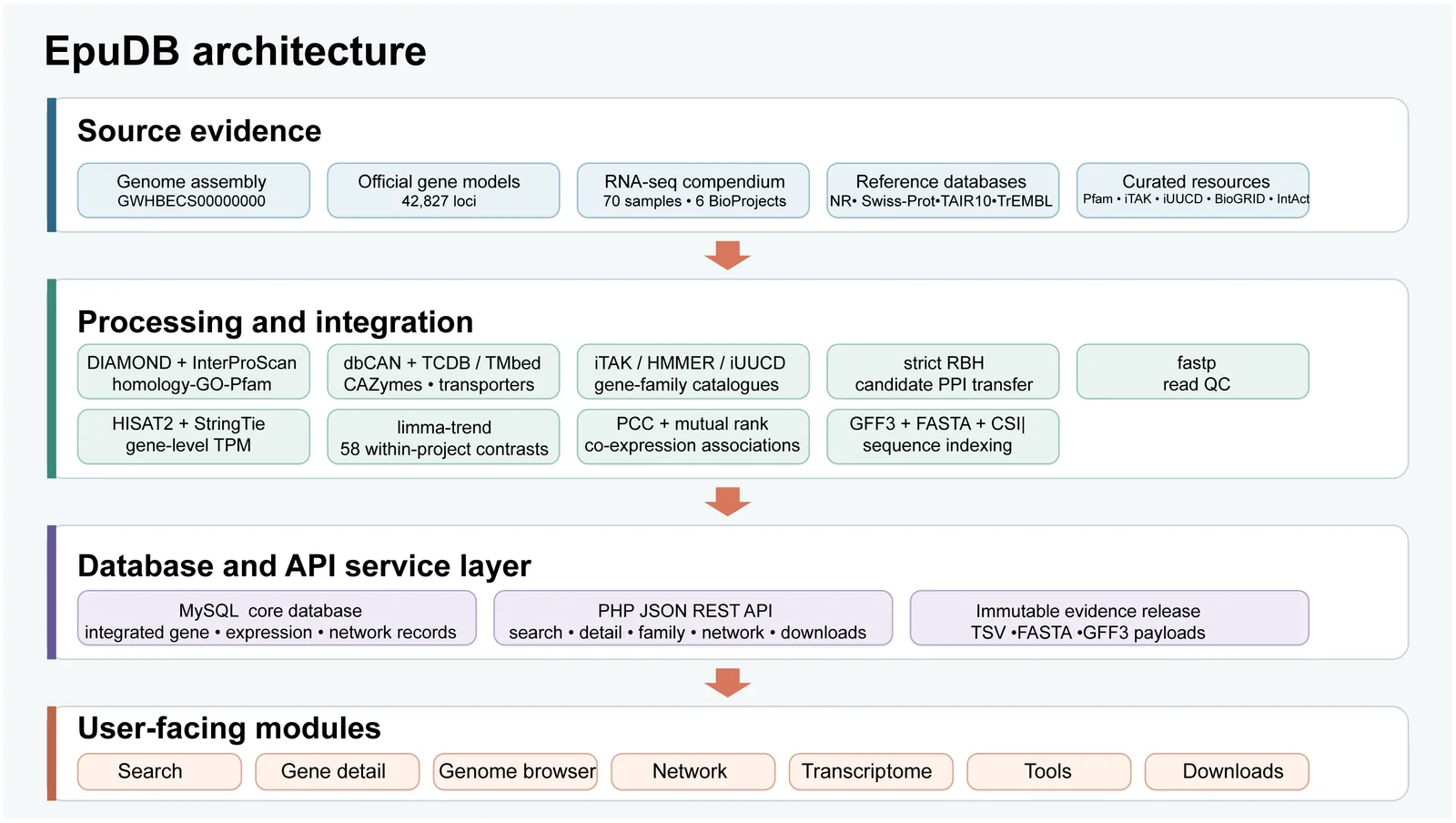
Architecture of EpuDB. Genome, annotation, gene-family, interaction and RNA-seq inputs feed a MySQL-backed portal and JSON REST API. Differential expression was calculated with limma-trend for all 58 within-project contrasts. Candidate PPI pairs are orthology-inferred, and co-expression edges represent associations rather than direct regulatory evidence.

A PHP REST API exposed these data through a uniform JSON envelope containing status, message, error_code, api_version, data_release and data. Fifteen documented endpoints covered database statistics, search, gene details, gene families, networks, sequences, sample and contrast metadata, DEG results, expression profiles, KEGG records, enrichment, multiple sequence alignment, primer design and bulk-download metadata.

All 15 documented API endpoints and four representative error paths passed local-deployment tests (19/19). Success and error responses used the API envelope and the database identifier EpuDB; unexpected failures returned a sanitized HTTP 500 payload. A descriptive comparison with five established plant resources covered genome, annotation, expression, analysis and download functions. The categories distinguish resource-specific scope and are not a superiority score.

The Network module provided separate visualizations and tables for the historical and BioProject-adjusted co-expression layers and for orthology-inferred candidate PPI relationships. Candidate PPI tables exposed the experimentally supported Arabidopsis source edge and its evidence provenance.

### 3.2 Integrated genome, annotation and gene-family resources

EpuDB used a 3,392,132,203-bp assembly comprising six nuclear pseudochromosomes, chloroplast and mitochondrial sequences. The nuclear pseudochromosomes ranged from 458.8 to 637.4 Mb. The database contained 42,827 annotated gene loci, whose structures were available through the embedded JBrowse2 browser (**Figure 2A**; **Table 2**).

**Figure 2:**
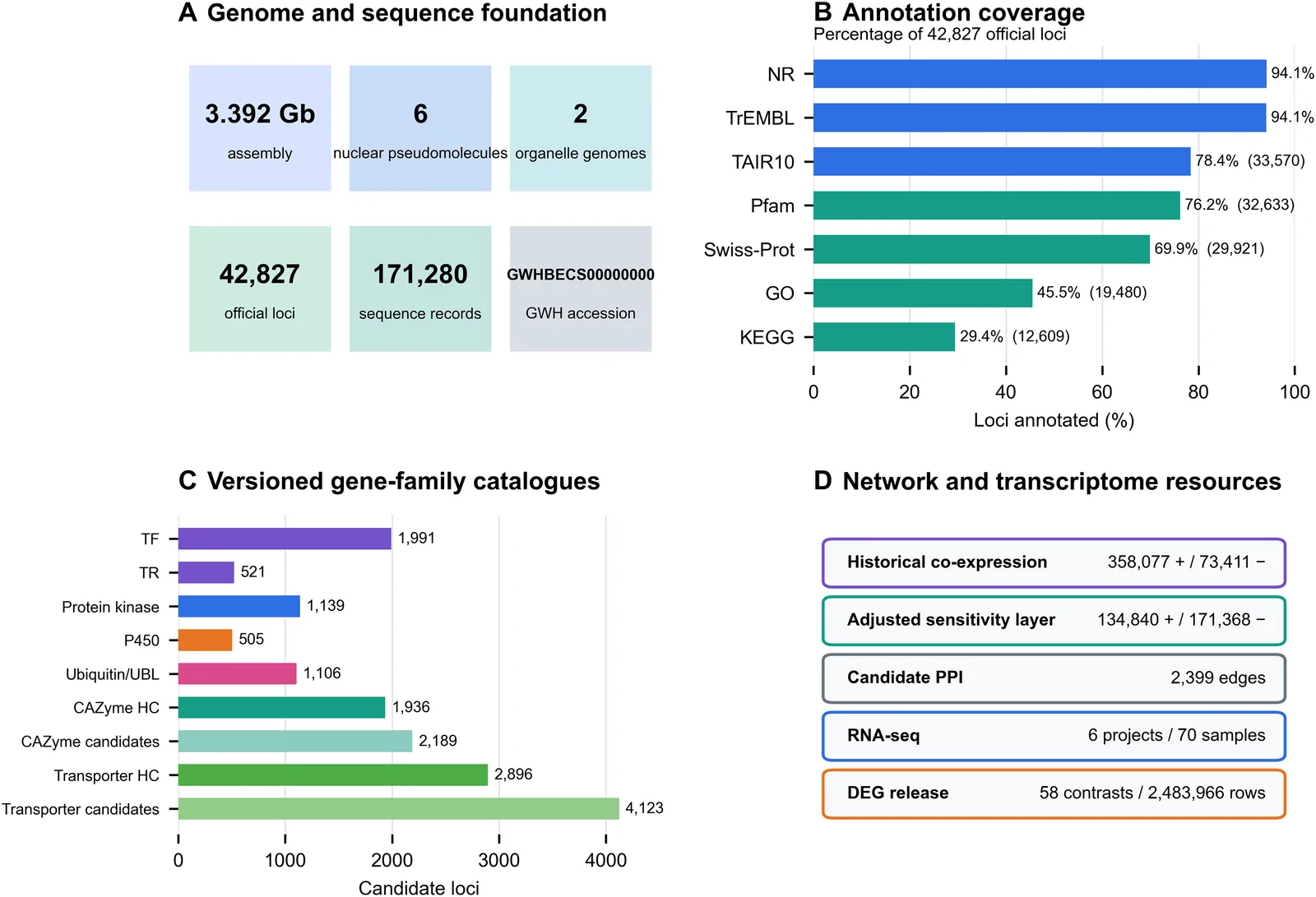
Data content and quality-aware summary of EpuDB. **(A)** Genome and sequence foundation. **(B)** Locus-level coverage of seven annotation sources. **(C)** Gene-family catalogues, with transcription factors and transcriptional regulators reported separately and CAZyme and transporter tiers kept distinct. **(D)** Historical and BioProject-adjusted co-expression layers, orthology-inferred candidate PPI pairs and transcriptome and DEG resources.

**Table 2:** Summary statistics of data types integrated in EpuDB.

Data category	Number/content
Official gene loci	42,827
Homology annotation records	144,106
NR	40,321 loci (94.15%)
TrEMBL	40,294 loci (94.09%)
TAIR10	33,570 loci (78.39%)
Pfam	32,633 loci (76.20%); 57,273 records
Swiss-Prot	29,921 loci (69.86%)
GO	19,480 loci (45.49%); 38,762 assignments
KEGG	12,609 loci (29.44%); 30,209 records
Transcription factors	1,991 genes; iTAK 2.0.7
Transcriptional regulators	521 genes; iTAK 2.0.7
Protein kinases	1,139 genes; iTAK 2.0.7
Cytochrome P450s	505 genes; PF00067.28/HMMER 3.4 --cut_ga
Ubiquitination-system components	1,106 genes; iUUCD2/HMMER 3.4
CAZyme/associated high-confidence tier	1,936 genes
CAZyme candidate tier	2,189 genes
Transporter-system high-confidence tier	2,896 genes
Transporter candidate tier	4,123 genes
Orthology-inferred candidate PPI pairs	2,399
Co-expression	358,077 positive; 73,411 negative
BioProject-adjusted sensitivity layer	134,840 positive; 171,368 negative
RNA-seq BioProjects/samples	6/70
DEG comparison groups	58 within-project contrasts
DEG results	2,483,966 all-gene contrast rows; 59,713 significant (30,067 up; 29,646 down)
Sequence records	171,280
EpuDB data files	52 payload files
REST API endpoints	15; 19/19 endpoint/error-path tests passed

Functional annotation produced 144,106 homology records. At least one record was available for 40,321 loci in NR (94.15%), 40,294 in TrEMBL (94.09%), 33,570 in TAIR10 (78.39%), 32,633 in Pfam (76.20%), 29,921 in Swiss-Prot (69.86%), 19,480 in GO (45.49%) and 12,609 in KEGG (29.44%). The denominator was the set of 42,827 official loci. The source tables contained 38,762 GO assignments, 57,273 Pfam-domain records and 30,209 KEGG/KO records (**Figure 2B**; **Table 2**).

The family catalogues contained 1,991 transcription factors, 521 transcriptional regulators and 1,139 protein kinases, 505 cytochrome P450 candidates from PF00067.28/HMMER 3.4 --cut_ga searches and 1,106 ubiquitination-system candidates from the iUUCD2 HMM library. Direct whole-proteome reannotation yielded 1,936 high-confidence CAZyme or CAZyme-associated proteins plus 2,189 candidates, and 2,896 high-confidence transporter-system proteins plus 4,123 candidates. High-confidence and candidate tiers remained separate in the database (**Figure 2C**).

### 3.3 Transcriptome and network resources

EpuDB integrated 70 RNA-seq samples from six public BioProjects. The analysis covered all 58 within-project contrasts and retained 42,827 rows per contrast, for 2,483,966 rows in total. Of these, 59,713 met adjusted P < 0.05 and |log_2_FC| ≥ 1, including 30,067 upregulated and 29,646 downregulated records. Ten contrasts were labelled power-limited because one or both groups contained fewer than three libraries. Low-expression loci remained downloadable with tested = false, null inferential statistics and filter_status = ‘Not tested—low expression’ (**Figure 2D**; **Tables 1**, **2**; **Supplementary Table S2**, **S3**). The annotation-coverage source table is supplied as **Supplementary Table S4**.

All 70 fastp and HISAT2 summaries matched the expression-matrix run accessions, and after-filter read totals reconciled with HISAT2 inputs for all 70 runs. Post-filter Q30 ranged from 91.34% to 97.66%, 98.66–100.00% of reads were retained and overall alignment rates ranged from 71.83% to 92.46%. BioProject median alignment rates ranged from 84.33% to 91.28%. Median gene-detection rates ranged from 37.17% to 45.87% at TPM ≥ 1 and from 48.11% to 59.49% at TPM > 0 (**Supplementary Table S5**). PC1 and PC2 explained 28.33% and 15.81% of the variance. BioProject was associated with expression structure (PERMANOVA pseudo-F = 9.659, R² = 0.4301, P = 0.0001), and dispersion also differed (PERMDISP pseudo-F = 41.686, P = 0.0001). We therefore describe BioProject-associated variation rather than a purely technical batch effect. No sample met the prespecified multi-metric outlier rule, so all 70 samples were retained (**Supplementary Figure S1**).

The historical co-expression layer contained 358,077 positive edges and 73,411 negative edges among 24,116 expression-eligible genes across the 70-sample TPM matrix. Positive edges used PCC > 0.70 and MR < 30, whereas negative edges used PCC < −0.50 and MR < 30. The BioProject-adjusted layer, generated by centering log_2_(TPM + 1) within each BioProject before correlation, contained 134,840 positive and 171,368 negative edges under the same thresholds. The two layers were retained as separate network resources, and adjusted edges carried within-project direction and leave-one-BioProject-out support annotations (**Figure 2D**; **Supplementary Figure S1**, **S3**).

The protein-interaction resource combined 12,003 evidence-filtered Arabidopsis source interactions with 1,901 strict reciprocal BLASTP best-hit mappings. Requiring accepted reciprocal best hits for both partners yielded 2,399 non-redundant orthology-inferred candidate PPI pairs involving 1,441 *E. pubescens* genes, a 90.44% reduction from the original 25,105-edge orthology-expanded network (**Supplementary Figure S1**, **S3**).

### 3.4 Case study: mining icariin-biosynthesis enzymes and their candidate regulators

EpuDB reconstructed the enzymatic backbone of icariin biosynthesis. KEGG/KO and homology searches recovered the upstream enzyme complement supplying kaempferol: PAL (2 loci), C4H/CYP73A (1 locus), 4CL (5 loci), CHS (14 loci), CHI (6 loci), F3H (2 loci) and FLS (15 loci) (**Figure 3A**; **Supplementary Table S1**). The reviewed downstream sequence ran from kaempferol through 8-prenylkaempferol, icaritin and baohuoside I to icariin. Prenylation, 4′-O-methylation, 3-O-rhamnosylation and 7-O-glucosylation were treated as separate enzymatic steps. This pathway census defined the enzyme set used for regulator mining.

Reference-enzyme retrieval provided locus-level mapping examples. The directly characterized *E. pubescens* prenyltransferase *EpPT8* was uniquely retrieved as Ebr02G069700, together with its annotation, PF01040 UbiA-family domain and expression profile. Ep7GT and EpPF3RT, characterized in *E. pseudowushanense*, were retained as cross-species homology candidates; their leading *E. pubescens* matches were Ebr04G027460.1 and Ebr06G041140.1, respectively (Shen et al., 2022; Feng et al., 2018, 2019).

For the prenylation branch, the search identified 12 loci annotated as flavonoid or aromatic prenyltransferases and 18 loci carrying PF01040; coordinate inspection highlighted two candidate arrays on pseudochromosome 2, including Ebr02G069700 in the six-gene region at 570.62–573.85 Mb (**Figure 3B**). For the O-methylation branch, five *E. pubescens* proteins carrying the PF00891 Methyltransf_2 O-methyltransferase domain were audited together with PF08100 dimerization architecture, homology, KO annotation, protein length and expression breadth. Ebr01G061770 and Ebr01G061810 were joint leading computational candidates and formed the O-methyltransferase arm of the three-gene case study, while Ebr01G061760, Ebr01G061820 and Ebr03G017960 remained secondary candidates (**Figure 3C**).

**Figure 3:**
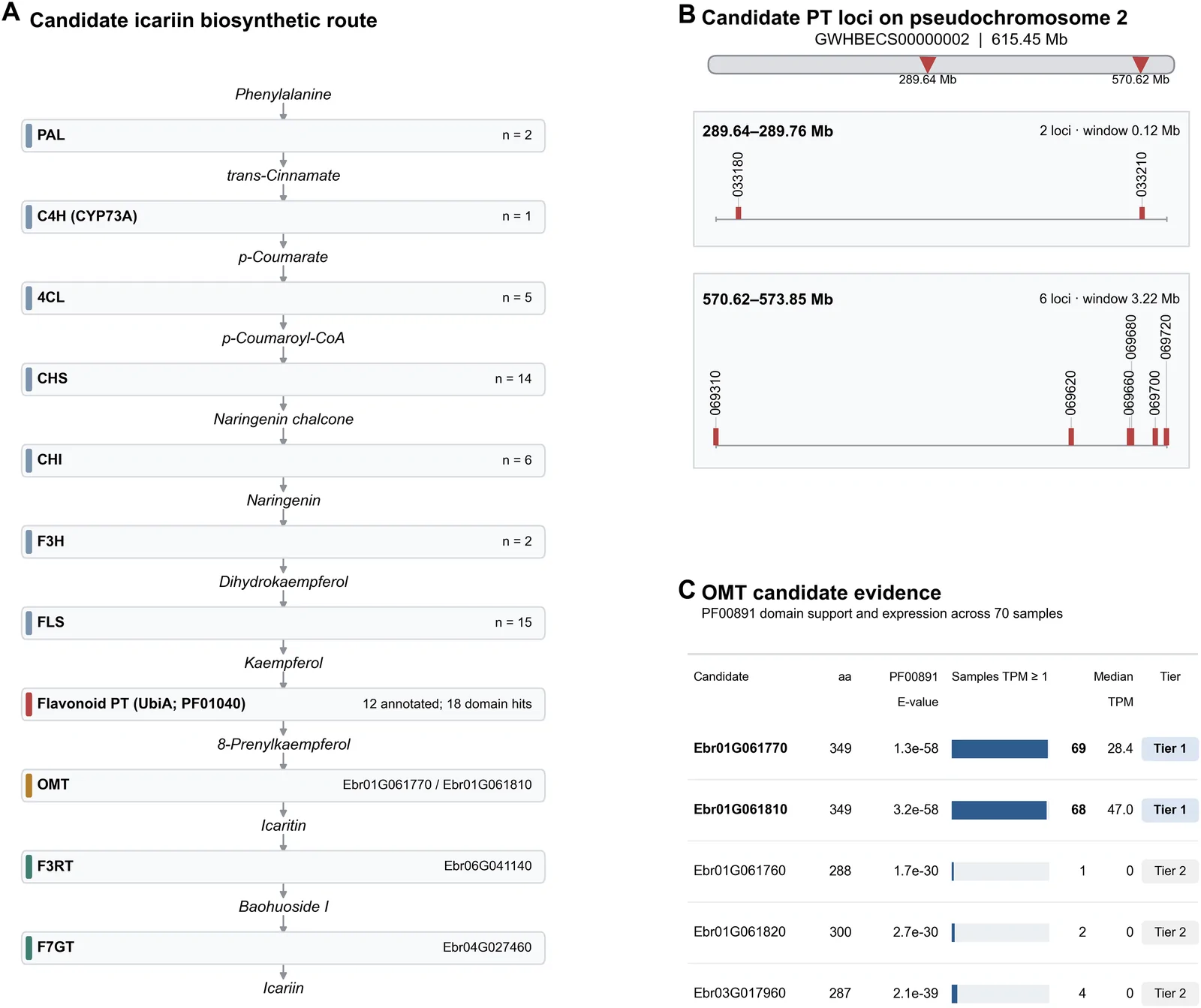
Icariin-pathway candidate discovery in EpuDB. **(A)** Candidate pathway reconstruction from upstream flavonoid enzymes and reviewed downstream reactions. **(B)** Candidate prenyltransferase loci on pseudochromosome 2, including the region containing Ebr02G069700. **(C)** Evidence for five OMT candidates carrying PF00891 (Methyltransf_2 O-methyltransferase) architecture, including protein length, domain E-value, the number of RNA-seq libraries with TPM ≥ 1, median TPM and evidence tier.

The third case-study locus, Ebr05G059870, was selected as the candidate regulator because it was the leading R2R3-MYB recovered by intersecting positive co-expression neighbors with the transcription-factor catalogue. In the historical network, it was associated with four prenyltransferase candidates: Ebr02G069310 (PCC = 0.824, MR = 3.5), Ebr02G069680 (PCC = 0.819, MR = 5.3), Ebr02G069620 (PCC = 0.811, MR = 9.2) and Ebr01G001570 (PCC = 0.769, MR = 8.5). Their BioProject-adjusted PCC/MR values were 0.713/41.28, 0.693/61.97, 0.638/142.03 and 0.665/92.37, respectively. Ebr05G059870 contains two PF00249 Myb-like DNA-binding domains. **Figures 4A, B** summarize the candidate’s domain and expression evidence, whereas **Figures 4C, D** show that adjusted associations and network degree depend on the prespecified thresholds. **Figure 4** and **Table 3** report these evidence layers separately. This choice is also consistent with prior transcriptome-based network analysis in *E. pubescens* (Xu et al., 2023).

**Figure 4:**
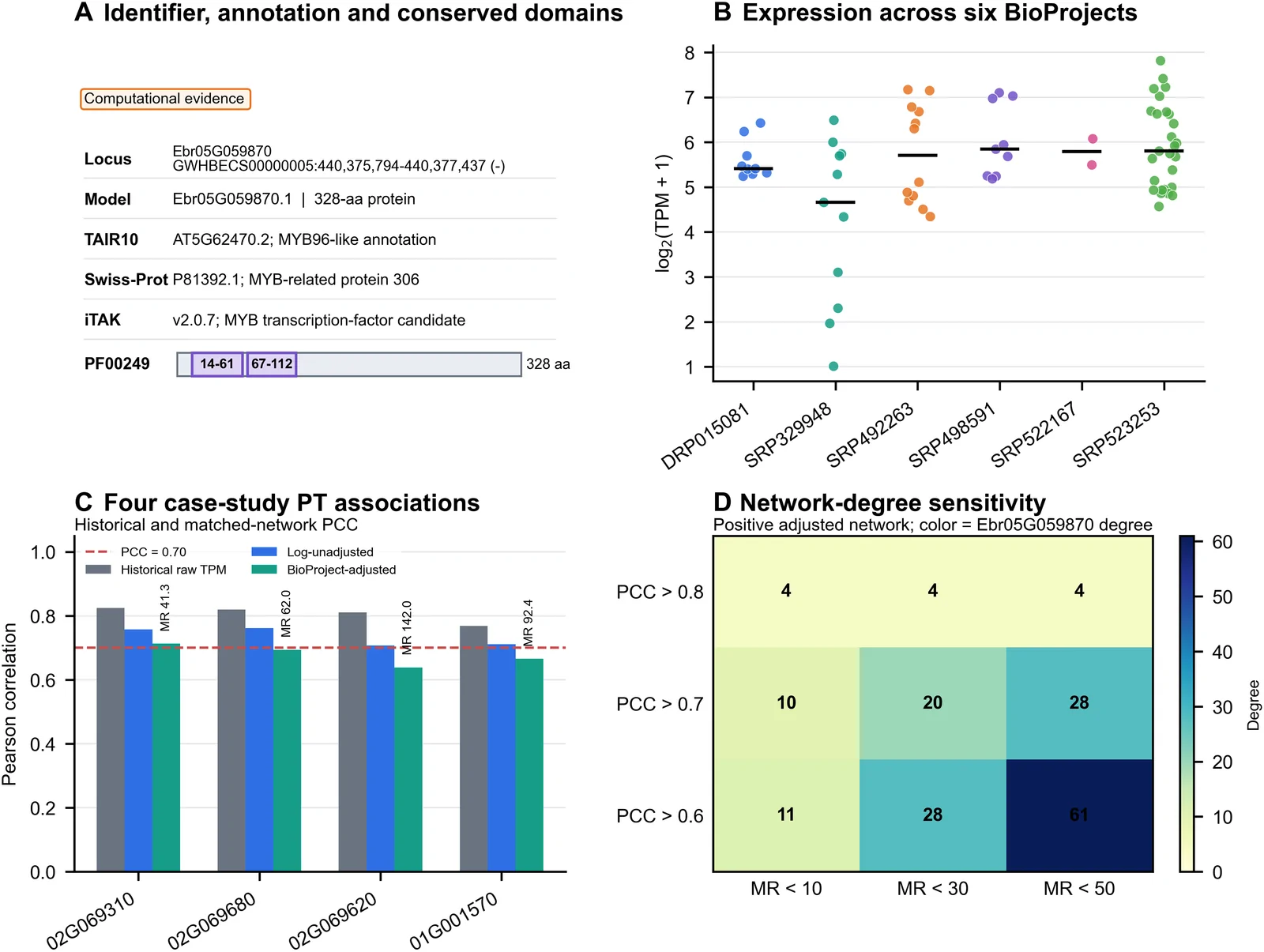
Evidence profile for the computationally prioritized Ebr05G059870 candidate. **(A)** Identifier, homology annotations, iTAK classification and two PF00249 MYB-like DNA-binding domains. **(B)** Expression across six BioProjects; points are individual public-library measurements from heterogeneous designs and are not interchangeable biological replicates. **(C)** Co-expression associations with prenyltransferase candidates compared with matched log-unadjusted and BioProject-adjusted PCC values; none passes both adjusted criteria (positive PCC > 0.70 and MR < 30). **(D)** Degree of Ebr05G059870 across the prespecified positive-network threshold grid.

**Table 3:** Candidate transcription factors linked to icariin-pathway genes in the historical EpuDB positive co-expression subset.

TF gene ID	TF family	*Arabidopsis* best hit (annotation)	Co-expressed current-pathway gene(s)	PCC	MR
*Ebr05G059870*	MYB	*AT5G62470* (*MYB96*)	*Ebr02G069310*, *Ebr02G069620*, *Ebr02G069680*, *Ebr01G001570* (PT candidates)	0.77–0.82	3.5–9.2
*Ebr06G045020*	C2C2-Dof	*AT4G38000* (Dof4.7)	*Ebr03G048960*, *Ebr02G068690* (*FLS*)	0.96–0.98	5.3–21.7
*Ebr01G027580*	bHLH	*AT2G20180* (PIF3-like 5)	*Ebr02G069620*, *Ebr02G069680* (PT candidates)	0.76–0.80	4.2–13.6
*Ebr05G012600*	C2C2-YABBY	*AT2G26580* (YABBY family)	*Ebr03G031750* (*CHI*), *Ebr03G053950* (PT candidate)	0.78–0.79	18.8–28.1
*Ebr04G002370*	MYB	*AT3G01140* (MYB106)	*Ebr03G053950* (PT candidate)	0.85	3.7
*Ebr01G033050*	MYB	*AT4G38620* (MYB4)	*Ebr01G026640* (*CHS*)	0.85	4.6

Database-wide annotation coverage and BioProject-level RNA-seq quality are summarized in **Figures 5A, B**. The positive-control audit retrieved Ebr02G069700 for the directly characterized *E. pubescens* prenyltransferase *EpPT8*. Cross-species controls returned sequence-supported *E. pubescens* homologs for *Ep7GT* and EpPF3RT from *E. pseudowushanense* and EsMYBF1 from *E. sagittatum* (Shen et al., 2022; Feng et al., 2018, 2019; Huang et al., 2016). These four controls were analyzed separately from the three-locus icariin case study (**Figure 5C**; **Supplementary Table S6**). The fixed-threshold network benchmark is summarized in **Supplementary Table S7**.

**Figure 5:**
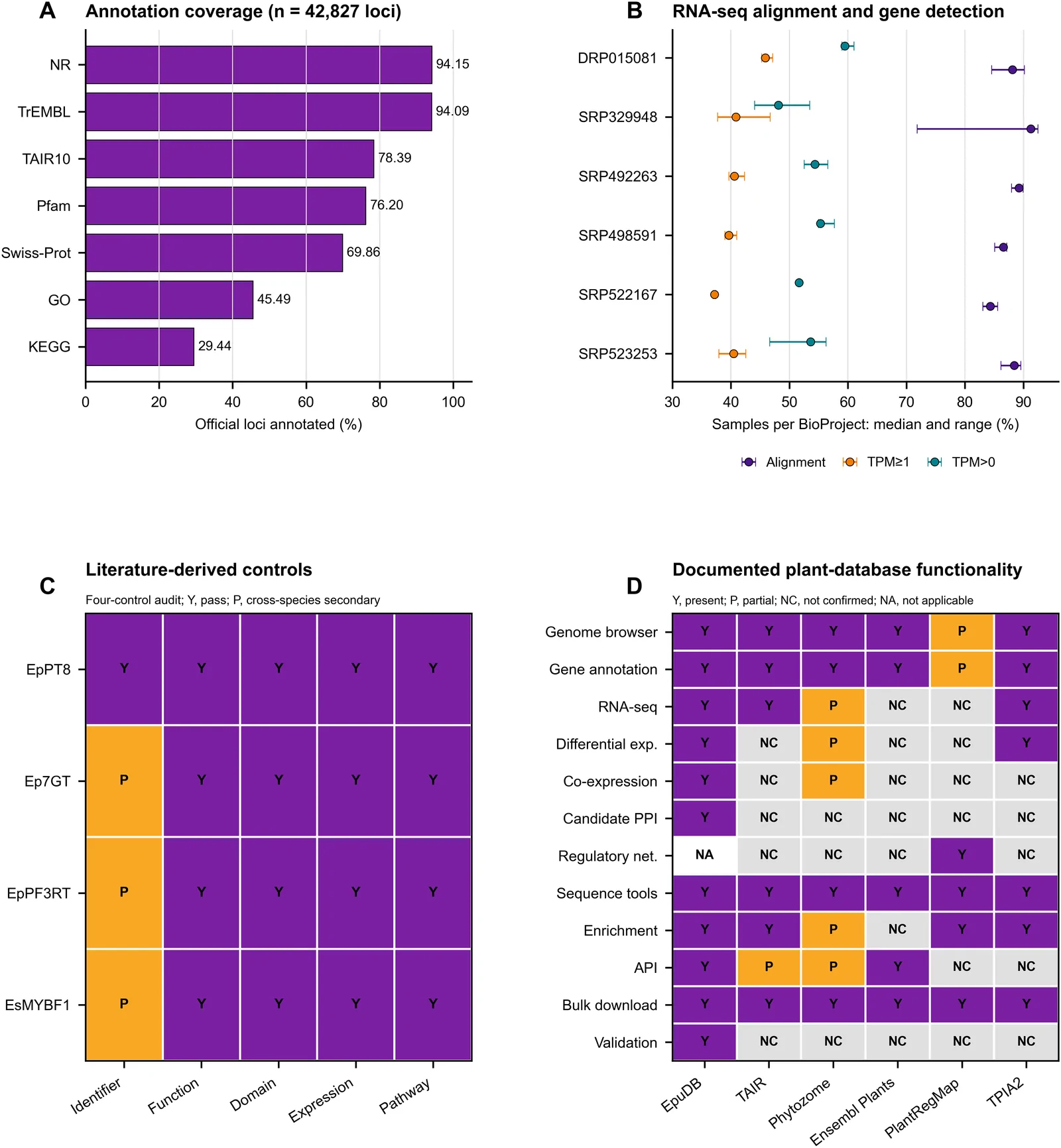
Validation and benchmarking of EpuDB. **(A)** Coverage of seven annotation sources. **(B)** BioProject-level RNA-seq mapping and gene-detection summaries. **(C)** Retrieval of four literature-derived controls used in the manuscript: EpPT8, Ep7GT, EpPF3RT and EsMYBF1. The panel distinguishes direct same-species retrieval from cross-species homology support. **(D)** Descriptive functionality comparison with five plant databases.

## 4 Discussion

### 4.1 Importance of EpuDB for gene discovery

EpuDB brings the main evidence layers for *E. pubescens* into one queryable framework. It links the chromosome-level gene catalogue to annotation, expression profiles, differential-expression results, co-expression layers, candidate PPI records and programmatic access. For prenylflavonoid biosynthesis, this organization matters because experimental studies, cross-species homology and computational associations are distributed across different sources. EpuDB keeps these evidence classes separate while allowing users to follow a pathway or regulator hypothesis to locus-level records. Its role is therefore to organize evidence and narrow candidates for testing, not to replace functional experiments.

### 4.2 Impact of BioProject adjustment on co-expression networks

Cross-project heterogeneity affects how co-expression edges should be interpreted. In the integrated expression matrix, BioProject was associated with expression structure (PERMANOVA pseudo-F = 9.659, R² = 0.4301, P = 0.0001), and dispersion also differed among projects (PERMDISP pseudo-F = 41.686, P = 0.0001). However, BioProject was confounded with tissue, developmental stage, treatment, time point and sequencing platform. We therefore did not apply a global ComBat correction, because a project-wide adjustment could remove biological differences encoded by the project variable. Instead, we retained the historical pooled network and added a BioProject-adjusted sensitivity layer by centering log_2_(TPM + 1) values within each BioProject before calculating PCC and MR on the same genes and samples. This changed the positive-edge count from 358,077 to 134,840 and the negative-edge count from 73,411 to 171,368, showing that edge density and the balance between positive and negative associations are sensitive to cross-project composition. Project-specific PCCs, within-project direction support and leave-one-BioProject-out analyses therefore serve as edge-level sensitivity annotations rather than proof of biological edge origin. Users should treat cross-project expression contrasts as descriptive and prioritize within-BioProject comparisons when inferring co-expression. In the Ebr05G059870 example, adjustment weakened the four historical associations and increased their MR values, so the candidate remains a computational priority for testing rather than a robustly validated regulator.

### 4.3 Comparison with related resources

The comparison with TAIR, Phytozome, Ensembl Plants, PlantRegMap and TPIA2 was descriptive. EpuDB combined genome browsing, gene search and annotation, RNA-seq exploration, DEG retrieval with test-status fields, co-expression, sequence and enrichment tools, bulk download, validation reporting and a documented API; API tests are documented in **Supplementary Table S8**. PlantRegMap specialized in regulatory networks, whereas the other resources differed in scope and emphasis. Presence, partial availability and not-confirmed categories describe documented functionality and are not numerical quality or superiority scores (**Figure 5D**; **Supplementary Table S9**). The CAZyme and transporter catalogues are supplied as **Supplementary Table S10**.

EpuDB also provides a species-specific resource for *E. pubescens*, where prenylflavonoid-biosynthesis genes are a central use case. The database connects the official chromosome-scale gene catalogue with functional annotations, curated gene families, DEG results, TPM profiles and orthology-inferred candidate PPI and co-expression networks. Users can inspect sequence, annotation, expression and network evidence within the same environment.

### 4.4 Limitations and future development

EpuDB is a hypothesis-generating resource. Gene-family HMM and classification calls, PF00891/PF08100 OMT architecture, CAZy families, TCDB homology and topology define candidate classes but do not establish exact biochemical activities, substrates, transport direction, localization or physiological roles. The five-gene OMT ranking and 18 PF01040-containing prenyltransferase loci therefore require biochemical validation. The absence of a public EpOMT4 sequence also prevented sequence-specific mapping. BioProject adjustment removes between-project means that include genuine biological differences, so it cannot isolate a purely technical batch effect. Project-specific correlations are descriptive, and SRP522167 contains only two samples, which is insufficient for an interpretable within-project correlation. Both co-expression layers represent association rather than direct regulation. Strict-RBH PPI transfer is likewise predictive: the experimental evidence belongs to Arabidopsis source interactions, not *E. pubescens* candidate edges. The small, partly cross-species control set cannot establish database-wide annotation, expression or network validity. Broader validation will require additional uniquely mapped, experimentally characterized same-species genes and independent expression measurements.

## 5 Conclusions

EpuDB integrates the official chromosome-level *E. pubescens* genome with multi-database annotation, gene-family catalogues, 2,399 orthology-inferred candidate PPI pairs, historical and BioProject-adjusted co-expression layers, a 70-sample TPM matrix and 58 within-project DEG contrasts containing 2,483,966 all-gene rows. Annotation coverage, RNA-seq quality, positive controls, network benchmarking, threshold sensitivity, API tests and plant-database functionality are reported with explicit evidence boundaries. The web portal and API support reproducible retrieval while keeping homology, prediction, correlation and experimental evidence distinct.

## Data Availability

The original contributions presented in the study are included in the article/**Supplementary Material**. Further inquiries can be directed to the corresponding authors.

## Supplementary material

Supplementary Figure 1 RNA-seq quality and BioProject-adjusted network audit. Read and mapping metrics, expression-space diagnostics, matched-network comparison and project-support analyses. BioProject is confounded with biological design and is treated as a composite source variable.

Supplementary Figure 2 Gene-centered EpuDB workflow. Query, evidence retrieval, downstream tools and hypothesis generation. Database output prioritizes candidates for experiments and does not establish mechanism.

Supplementary Figure 3 Threshold sensitivity of the BioProject-adjusted network. **(A, B)** Positive- and negative-edge counts across PCC and MR grids. **(C)** Jaccard overlap of positive configurations with the network. **(D)** Fraction of negative edges retained above the matching PCC threshold in all six leave-one-BioProject-out analyses.

## References

[B1] Aramaki T. Blanc-Mathieu R. Endo H. Ohkubo K. Kanehisa M. Goto S. . (2020). KofamKOALA: KEGG ortholog assignment based on profile HMM and adaptive score threshold. Bioinformatics 36, 2251–2252. doi: 10.1093/bioinformatics/btz859 PMC7141845 31742321

[B2] Bernhofer M. Rost B. (2022). TMbed: transmembrane proteins predicted through language model embeddings. BMC Bioinf. 23, 326. doi: 10.1186/s12859-022-04873-x 35941534 PMC9358067

[B3] Buchfink B. Reuter K. Drost H. G. (2021). Sensitive protein alignments at tree-of-life scale using DIAMOND. Nat. Methods 18, 366–368. doi: 10.1038/s41592-021-01101-x 33828273 PMC8026399

[B4] Camacho C. Coulouris G. Avagyan V. Ma N. Papadopoulos J. Bealer K. . (2009). BLAST+: architecture and applications. BMC Bioinf. 10, 421. doi: 10.1186/1471-2105-10-421 20003500 PMC2803857

[B5] Chen M. Ma Y. Wu S. Zheng X. Kang H. Sang J. . (2021). Genome Warehouse: a public repository housing genome-scale data. Genomics Proteomics Bioinf. 19, 584–589. doi: 10.1016/j.gpb.2021.04.001 34175476 PMC9039550

[B6] Chen S. Zhou Y. Chen Y. Gu J. (2018). fastp: an ultra-fast all-in-one FASTQ preprocessor. Bioinformatics 34, i884–i890. doi: 10.1093/bioinformatics/bty560 30423086 PMC6129281

[B7] Deng W. Nickle D. C. Learn G. H. Maust B. Mullins J. I. (2007). ViroBLAST: a stand-alone BLAST web server for flexible queries of multiple databases and user's datasets. Bioinformatics 23, 2334–2336. doi: 10.1093/bioinformatics/btm331 17586542

[B8] Diesh C. Stevens G. J. Xie P. De Jesus Martinez T. Hershberg E. A. Leung A. . (2023). JBrowse 2: a modular genome browser with views of synteny and structural variation. Genome Biol. 24, 74. doi: 10.1186/s13059-023-02914-z 37069644 PMC10108523

[B9] Edgar R. C. (2004). MUSCLE: multiple sequence alignment with high accuracy and high throughput. Nucleic Acids Res. 32, 1792–1797. doi: 10.1093/nar/gkh340 15034147 PMC390337

[B10] Feng K. Chen R. Xie K. Chen D. Guo B. Liu X. . (2018). A regiospecific rhamnosyltransferase from *Epimedium pseudowushanense* catalyzes the 3-O-rhamnosylation of prenylflavonols. Org. Biomol. Chem. 16, 452–458. doi: 10.1039/C7OB02763J 29265155

[B11] Feng K. Chen R. Xie K. Chen D. Liu J. Du W. . (2019). Ep7GT, a glycosyltransferase with sugar donor flexibility from *Epimedium pseudowushanense*, catalyzes the 7-O-glycosylation of baohuoside. Org. Biomol. Chem. 17, 8106–8114. doi: 10.1039/C9OB01352K 31455963

[B12] Huang W. Khaldun A. B. Chen J. Zhang C. Lv H. Yuan L. . (2016). A R2R3-MYB transcription factor regulates the flavonol biosynthetic pathway in a traditional Chinese medicinal plant, *Epimedium sagittatum*. Front. Plant Sci. 7, 1089. doi: 10.3389/fpls.2016.01089 27493658 PMC4954812

[B13] Huang Z. Li M. Li A. Wei G. Lin X. Jia L. . (2026). *Epimedium*: medicinal plant resources, chemical constituents, pharmacological actions, and biosynthesis of prenylated flavonol glycosides. Ind. Crops Prod. 245, 123320. doi: 10.1016/j.indcrop.2026.12332042574925

[B14] Jones P. Binns D. Chang H. Y. Fraser M. Li W. McAnulla C. . (2014). InterProScan 5: genome-scale protein function classification. Bioinformatics 30, 1236–1240. doi: 10.1093/bioinformatics/btu031 24451626 PMC3998142

[B15] Kerrien S. Aranda B. Breuza L. Bridge A. Broackes-Carter F. Chen C. . (2012). The IntAct molecular interaction database in 2012. Nucleic Acids Res. 40, D841–D846. doi: 10.1093/nar/gkr1088 22121220 PMC3245075

[B16] Kim D. Paggi J. M. Park C. Bennett C. Salzberg S. L. (2019). Graph-based genome alignment and genotyping with HISAT2 and HISAT-genotype. Nat. Biotechnol. 37, 907–915. doi: 10.1038/s41587-019-0201-4 31375807 PMC7605509

[B17] Langfelder P. Horvath S. (2008). WGCNA: an R package for weighted correlation network analysis. BMC Bioinf. 9, 559. doi: 10.1186/1471-2105-9-559 19114008 PMC2631488

[B18] Li B. Lima M. R. M. Nie Y. Xu L. Liu X. Yuan H. . (2021). HPLC-DAD fingerprints combined with multivariate analysis of Epimedii Folium from major producing areas in Eastern Asia: Effect of geographical origin and species. Front. Pharmacol. 12, 761551. doi: 10.3389/fphar.2021.761551 34899314 PMC8662750

[B19] Obayashi T. Kinoshita K. (2009). Rank of correlation coefficient as a comparable measure for biological significance of gene coexpression. DNA Res. 16, 249–260. doi: 10.1093/dnares/dsp016 19767600 PMC2762411

[B20] Pertea M. Pertea G. M. Antonescu C. M. Chang T. C. Mendell J. T. Salzberg S. L. (2015). StringTie enables improved reconstruction of a transcriptome from RNA-seq reads. Nat. Biotechnol. 33, 290–295. doi: 10.1038/nbt.3122 25690850 PMC4643835

[B21] Ritchie M. E. Phipson B. Wu D. Hu Y. Law C. W. Shi W. . (2015). limma powers differential expression analyses for RNA-sequencing and microarray studies. Nucleic Acids Res. 43, e47. doi: 10.1093/nar/gkv007 25605792 PMC4402510

[B22] Saier M. H. Jr. Reddy V. S. Moreno-Hagelsieb G. Hendargo K. J. Zhang Y. Iddamsetty V. . (2021). The transporter classification database (TCDB): 2021 update. Nucleic Acids Res. 49, D461–D467. doi: 10.1093/nar/gkaa1004 33170213 PMC7778945

[B23] Shen G. Luo Y. Yao Y. Meng G. Zhang Y. Wang Y. . (2022). The discovery of a key prenyltransferase gene assisted by a chromosome-level *Epimedium pubescens* genome. Front. Plant Sci. 13, 1034943. doi: 10.3389/fpls.2022.1034943 36452098 PMC9702526

[B24] Stark C. Breitkreutz B. J. Reguly T. Boucher L. Breitkreutz A. Tyers M. (2006). BioGRID: a general repository for interaction datasets. Nucleic Acids Res. 34, D535–D539. doi: 10.1093/nar/gkj109 16381927 PMC1347471

[B25] Sze S. C. Tong Y. Ng T. B. Cheng C. L. Cheung H. P. (2010). Herba Epimedii: anti-oxidative properties and its medical implications. Molecules 15, 7861–7870. doi: 10.3390/molecules15117861 21060294 PMC6259234

[B26] Untergasser A. Cutcutache I. Koressaar T. Ye J. Faircloth B. C. Remm M. . (2012). Primer3--new capabilities and interfaces. Nucleic Acids Res. 40, e115. doi: 10.1093/nar/gks596 22730293 PMC3424584

[B27] Van Bel M. Diels T. Vancaester E. Kreft L. Botzki A. Van de Peer Y. . (2018). PLAZA 4.0: an integrative resource for functional, evolutionary and comparative plant genomics. Nucleic Acids Res. 46, D1190–D1196. doi: 10.1093/nar/gkx1002 PMC5753339 29069403

[B28] Wang D. Fan W. Guo X. Wu K. Zhou S. Chen Z. . (2020). MaGenDB: a functional genomics hub for Malvaceae plants. Nucleic Acids Res. 48, D1076–D1084. doi: 10.1093/nar/gkz953 PMC7145696 31665439

[B29] Wang L. Li Y. Guo Y. Ma R. Fu M. Niu J. . (2016). Herba Epimedii: an ancient Chinese herbal medicine in the prevention and treatment of osteoporosis. Curr. Pharm. Des. 22, 328–349. doi: 10.2174/1381612822666151112145907 26561074

[B30] Xu C. Liu X. Shen G. Fan X. Zhang Y. Sun C. . (2023). Time-series transcriptome provides insights into the gene regulation network involved in the icariin-flavonoid metabolism during the leaf development of *Epimedium pubescens*. Front. Plant Sci. 14, 1183481. doi: 10.3389/fpls.2023.1183481 37377796 PMC10291196

[B31] Zheng J. Ge Q. Yan Y. Zhang X. Huang L. Yin Y. (2023). dbCAN3: automated carbohydrate-active enzyme and substrate annotation. Nucleic Acids Res. 51, W115–W121. doi: 10.1093/nar/gkad328 37125649 PMC10320055

[B32] Zheng Y. Jiao C. Sun H. Rosli H. G. Pombo M. A. Zhang P. . (2016). iTAK: a program for genome-wide prediction and classification of plant transcription factors, transcriptional regulators, and protein kinases. Mol. Plant 9, 1667–1670. doi: 10.1016/j.molp.2016.09.014 27717919

[B33] Zhou J. Xu Y. Lin S. Guo Y. Deng W. Zhang Y. . (2018). iUUCD 2.0: an update with rich annotations for ubiquitin and ubiquitin-like conjugations. Nucleic Acids Res. 46, D447–d453. doi: 10.1093/nar/gkx1041 PMC5753239 29106644

